# The second wave: estimating the hidden asymptomatic prevalence of COVID-19 in Ireland as we plan for imminent immunisation

**DOI:** 10.12688/hrbopenres.13206.2

**Published:** 2022-02-25

**Authors:** Catherine Comiskey, Anne Snel, Prakashini Banka

**Affiliations:** 1 School of Nursing and Midwifery, Trinity College Dublin, The University of Dublin, Dublin, 2, Ireland

**Keywords:** COVID-19, prevalence, age, mixing, mitigation, lockdown, back-calculation, back-projection

## Abstract

Since the first case of COVID-19 in Ireland was recorded policy makers have introduced mitigation measures to control the spread of infection. Infection is spread by both known cases and hidden, undetected asymptomatic cases.  Asymptomatic individuals are people who transmit the virus but display no clinical symptoms. Current evidence reveals that this population is a major contributing factor to the spread of the disease. There is little or no knowledge of the scale of the hidden prevalence of all infections both asymptomatic and symptomatic in Ireland. Furthermore, as governments plan for the roll out of imminent immunisation programmes, the need to know the scale of the hidden prevalence and hence knowledge of the level of immunisation required is essential.

We describe and analyse the numbers of reported cases of COVID-19 in Ireland from the first case in February 2020 to mid-December 2020. Using the method of back-calculation we provide estimates of the asymptomatic prevalence of cases from June to December 2020.

The descriptive analysis highlighted two epidemic waves of known cases in the time period. Wave two from June to December included twice as many cases as wave one and cases were significantly younger. The back-calculation estimates of asymptomatic prevalence during this time period revealed that for every case known there was an additional unknown case and total prevalence in wave two was estimated to be approximately 95,000 as opposed to the reported 48,390 cases.

As prevalence in wave two is known to be spreading within and from younger age groups the role of mixing patterns on spread needs to be disseminated to the wider public to adequately inform them how personal modifications in behaviour can contribute to the control of the epidemic. While universally imposed lockdowns and mitigation measures may be essential, personal behavioural mixing choices are powerful protectors.

## Introduction

Coronavirus disease 2019 or COVID-19 is a novel human respiratory disease caused by the SARS-CoV-2 virus and was first identified in 2019 ^1^. The surveillance of COVID-19 cases in Ireland was integrated into the existing national Computerised Infectious Disease Reporting (CIDR) system when the notification of the disease was made mandatory in February 2020 ^2^. Since the first case of COVID-19 in Ireland was recorded policy makers have introduced mitigation measures to control the spread of infection ^3^. These measures included public health advice to stay and work at home, restrictions on travel, the closure of educational settings, the cancellation of routine hospital procedures and the isolation and contract tracing of cases identified through testing centres ^3^. It has been observed that during these periods of increased and subsequent decreased mitigation measures the reported number of positive cases has decreased and increased in line with the implementation and removal of the measures. These increases and decreases are referred to as epidemic waves ^4^ and their relationship to the mitigation measures have been clearly established and modelled in Ireland ^5^.

Infection we know is spread by both known cases and hidden, undetected asymptomatic cases. Asymptomatic individuals in the context of COVID-19 are people who are carriers of the virus but display no clinical symptoms. Current evidence reveals that this population is a major contributing factor to the spread of the disease, while escaping detection by public health surveillance systems ^5^. As a result of this lack of detection public health systems can record only the daily incidence of new known cases and there is little or no knowledge of the actual scale of the hidden cumulative prevalence of all infections both asymptomatic and symptomatic. Furthermore, as governments plan for the roll out of imminent national immunisation programmes, the need to know the scale of the hidden prevalence and hence knowledge of the level of immunisation required is essential to produce the so called ‘herd immunity’ defined as ‘the protection of populations from infection which is brought about by the presence of immune individuals’ ^5, 6^.

The aim of this research was to build on previous modelling work and provide an estimate of the hidden and asymptomatic prevalence of COVID-19 in Ireland during the second wave of infection from October to December 2020. Methods while developed nationally are applicable globally. The objectives were to provide a descriptive and comparative analysis of the first and second waves; to use the back-calculation method to provide an estimate of total prevalence of cases during the second wave and an estimate of the ratio of unknown asymptomatic cases to known symptomatic recorded cases and finally to provide recommendations for future research to enable effective immunisation modelling and planning.

## Methods

A plot of the five-day moving average of the reported numbers of COVID-19 cases from the first recorded case on the 29 ^th^ of February 2020 to the 8 ^th^ of December 2020 was prepared. Descriptive statistics illustrating the numbers of known cases, hospitalised cases, intensive care cases and deaths during this period were computed and cumulate cases by age group were derived. The Chi-squared test of association was used to test the independence of the relationship between the number of cases during an epidemic wave and the numbers of cases reported by age group. This statistic was also used to test the relationship between the number of cases during an epidemic wave the number of hospitalised cases by age group.

Following the statistical analysis of the known cases from the Irish reporting system the back-calculation method was implemented to estimate the numbers of asymptomatic and unknown cases. Working with observed symptomatic cases and the known incubation period, these models predict backwards in time through the incubation period distribution the total numbers of infected and asymptomatic cases these observed cases arose from.

The method of back-calculation also known as back-projection is well documented and implemented internationally for a wide variety of infectious and social epidemics, from HIV/AIDS to bio-terrorism to heroin use ^7– 10^. Previous use of the back-calculation model to predict the incidence and prevalence of disease, particularly AIDS, in the United States, the United Kingdom and Ireland is well documented ^11– 14^. The method is known as an indirect method and working with observed symptomatic cases and the known incubation period, the model predicts minimum estimates of the hidden numbers of infected cases. The model is given by,

$$C_{T}(t)=\int_{0}^{t}C_{U}(t-s)f(s)ds$$

Where *C _T_* ( *t*) describes the change in the incidence of the treated and known cases over a defined time period, *f*( *s*) is the incubation period distribution of the disease and *C _U_* ( *t*) is the unknown number of cases at time *t* we wish to solve for. The prevalence of the unknown cases over the defined time period is then given by,

$$\int_{0}^{t}C_{U}(t)dt$$

Given varying forms in the growth of the known cases *C _T_* ( *t*) and the incubation period *f*( *s*), the back-calculation model can be solved analytically as in Comiskey ^7, 8^, Comiskey and Hay ^15^, Dempsey and Comiskey ^10, 16^ or numerically as in Comiskey and Ruskin ^13^. The details of the incubation period distribution for COVID-19 *f*( *t*) are provided by Banka and Comiskey ^17^ who in their international scoping review found a mean incubation period of 6.7 days with a standard deviation of 4.0 days. The mathematical solution of the back-calculation equation when *f*( *t*) is described by the Gamma distribution as identified by Banka and Comiskey (2020) and given by Γ( *α*, *λ*) when *α* = 6 and when *α* = 3 were originally provided by Dempsey and Comiskey ^10, 16^ and latterly for early COVID-19 modelling in Ireland by Comiskey, Snel and Banka ^18^. These solutions were implemented here with data on daily COVID-19 cases reported to the national Computerised Infectious Disease Reporting (CIDR) system and available at website https://covid-19.geohive.ie/datasets/d8eb52d56273413b84b0187a4e9117be_0

This research received ethical approval from the Faculty of Health Sciences at Trinity College Dublin, The University of Dublin, Ireland.

## Results

**Figure 1.**
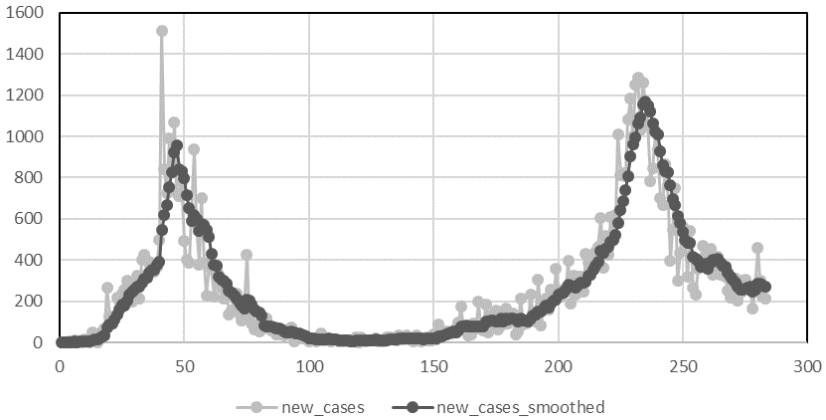
Daily reported incidence of COVID-19 from 29th February to 8th December 2020.

A plot of the number of daily cases reported to the national system from the 29 ^th^ of February to the 8 ^th^ December 2020 are provided in Figure 1. From this we can clearly see that Ireland has recorded to date two epidemic waves each with an increasing and decreasing phase. As two epidemic waves were observed in the data, we study each wave separately. To improve the fit of the models to the data we fit the models separately to the increasing and decreasing part of the wave.

From the reported data we can see that during wave one and two a total of 74,439 cases were reported and perhaps more importantly we can also see that greater numbers of individuals were infected within wave two. A total of 25,189 cases were reported in wave one and this almost doubled and increased to 49,250 cases being reported in the second wave. A comparison of the reported cases by age distribution across the two waves is provided in Table 1.

**Table 1.** COVID-19 cases in Ireland during the increasing and deceasing phases of epidemic wave 1 and wave 2, where the age of the case was known.

	Wave 1		Wave 2	
	Phase 1	Phase 2	Phase 1	Phase 2
	29 ^th^ Feb– 16 ^th^ Apr	17 ^th^ Apr – 10 ^th^ Jun	11 ^th^ Jun – 21 ^st^ Oct	22 ^nd^ Oct – 8 ^th^ Dec
**Total** **Cumulative** **Cases**	**12,405**	**25,189**	**52,226**	**74,439**
< 5 Years	72	161	1,013	1,972
5 – 14 Years	124	320	2,404	4,847
15 – 24 Years	809	1,861	8,667	12,976
25 – 34 Years	2,145	4,223	9,068	12,498
35 – 44 Years	2,315	4,438	8,370	11,673
45 – 54 Years	2,395	4,535	8,134	11,227
55 – 64 Years	1,806	3,232	5,841	7,935
> 65 Years	2,739	6,419	8,729	11,311
**Total Cases** **Within the** **Time Period**	**12,405**	**12,784**	**27,037**	**22,213**
< 5 Years	72	89	852	959
5 – 14 Years	124	196	2,084	2,443
15 – 24 Years	809	1,052	6,806	4,309
25 – 34 Years	2,145	2,078	4,845	3,430
35 – 44 Years	2,315	2,123	3,932	3,303
45 – 54 Years	2,395	2,140	3,599	3,093
55 – 64 Years	1,806	1,426	2,609	2,094
> 65 Years	2,739	3,680	2,310	2,582
**Hospitalised** **Cumulative** **Cases**	**2,026**	**3,321**	**4,055**	**5,343**
< 5 Years	13	20	42	58
5 – 14 Years	4	17	36	51
15 – 24 Years	43	71	123	171
25 – 34 Years	132	198	261	333
35 – 44 Years	164	260	327	424
45 – 54 Years	298	445	528	668
55 – 64 Years	308	489	605	765
> 65 Years	1,062	1,819	2,130	2,871
**Hospitalised** **Cases Within** **the Time** **Period**	**2,026**	**1,295**	**733**	**1,289**
< 5 Years	13	7	22	16
5 – 14 Years	4	13	19	15
15 – 24 Years	43	28	52	48
25 – 34 Years	132	66	63	72
35 – 44 Years	164	96	67	97
45 – 54 Years	298	147	83	140
55 – 64 Years	308	181	116	160
> 65 Years	1,062	757	311	741
**ICU** **Cumulative** **Cases**	**284**	**411**	**519**	**630**
**ICU Cases** **Within the** **Time Period**	284	127	108	111
**Healthcare** **Workers** **Cumulative** **Cases**	**3,090**	**8,099**	**10,012**	**12,302**
**Healthcare** **Workers** **Cases Within** **the Time** **Period**	3,090	5,009	1,913	2,290
**Total** **Cumulative** **Deaths**	**486**	**1,695**	**1,868**	**2,097**
**Total Deaths** **Within the** **Time Period**	486	1,209	173	229

A comparison of the distribution of known cases by age between wave one and wave two is provided in Table 2. We can see that there was a significant change in the age distribution of cases between the two time periods. Within wave one those aged over 65 years of age accounted for approximately between one fifth and one quarter of all reported cases while those under the age of 25 years approximately accounted for one tenth of all cases. Within wave two however this situation reversed with those over the age of 65 accounting for approximately one tenth of all cases and those under the age of 25 accounting for one third of all cases. 

**Table 2.** Proportions of cases by age within each epidemic wave and Chi-square results.

	Wave 1		Wave 2		*χ* ^2^
	Phase 1	Phase 2	Phase 1	Phase 2	
	29 ^th^ Feb– 16 ^th^ Apr	17 ^th^ Apr – 10 ^th^ Jun	11 ^th^ Jun – 21 ^st^ Oct	22 ^nd^ Oct – 8 ^th^ Dec	
**Cases**					
< 5 Years	0.58	0.70	3.15	4.32	*χ* ^2^ = 32.57 **p ≤ 0.001** df = 21
5 – 14 Years	1.00	1.53	7.71	11.00	
15 – 24 Years	6.52	8.23	25.17	19.40	
25 – 34 Years	17.29	16.25	17.92	15.44	
35 – 44 Years	18.66	16.61	14.54	14.87	
45 – 54 Years	19.31	16.74	13.31	13.92	
55 – 64 Years	14.56	11.15	9.65	9.43	
> 65 Years	22.08	28.79	8.54	11.62	
**Hospitalised** **Cases**					
< 5 Years	0.64	0.54	3.00	3.00	*χ* ^2^ = 32.57 **p ≤ 0.001** df = 21
5 – 14 Years	0.20	1.00	2.59	2.59	
15 – 24 Years	2.12	2.16	7.09	7.09	
25 – 34 Years	6.52	5.10	8.59	8.59	
35 – 44 Years	8.10	7.41	9.14	9.14	
45 – 54 Years	14.72	11.35	11.32	11.32	
55 – 64 Years	15.22	13.98	15.83	15.83	
> 65 Years	52.47	58.46	42.43	42.43	

Clearly the dynamics of spread changed in wave two as societal mitigation measures were relaxed and prevention measures within older person settings were enhanced. Exploring wave two in more detail using the back-calculation method we initially fitted, using simple regression techniques, separate curves *C _T_* ( *t*) to all of the known cases of COVID-19 during both the increasing and decreasing phase of wave two. This included cases where the age was unknown. These curves included exponential, logarithmic, quadratic and cubic models. Details of the best fitting curves amongst all curves fitted are provided in Table 3.

**Table 3.** *C _T_* ( *t*) details of the best fitting curve amongst all curves fitted to the known number of COVID-19 cases within wave two of the epidemic.

Epidemic Wave	Best fitting curve	R squared	F; df1, df2; p
Wave 2, increasing, 11 ^th^ June to 21 ^st^ October	*C _T_* ( *t*) = 0·120 *e* ^0·037 *t*^	0·953	2645.790; 1, 131; *p* ≤0·001
Wave 2, decreasing, 22 ^nd^ October to December 8 ^th^	*C _T_* ( *t*) = 0.622 *t* ^2^ – 341.160t + 47039.209	0.971	749.348; 2, 45; *p* ≤0·001

The solutions provided by Comiskey, Snel and Banka ^18^ for the unknown number of cases *C _U_* ( *t*) given the best fitting curve *C _T_* ( *t*) were then applied and the results are provided in Table 4. From Table 4, we can see that regardless of the exact nature of the Gamma distribution chosen for the incubation period, the model predicts that for each known infectious case reported there exists approximately one unreported asymptomatic infectious case contributing to infection within the population.

**Table 4.** Estimates of the hidden prevalence of COVID-19 in Ireland during the second epidemic wave.

Epidemic Wave Given *α* = 3	Estimated Hidden Prevalence, *C _U_* ( *t*)	Known Diagnosed Prevalence, *C _T_* ( *t*)	Estimated Total Prevalence	Ratio of Unknown to Known Cases
Wave 2, increasing phase *11 ^th^ June – 21 ^st^ October*	28,155	28,184	56,339	1.00:1
Wave 2, decreasing phase *22 ^nd^ October–8 ^th^ December*	18,764	20,206	38,970	0.93:1
**Total *11 ^th^ June – 8 ^th^*** ***December***	**46,919**	**48,390**	**95,309**	**0.97:1**
**Epidemic Wave** **Given *α* = 2**				
Wave 2, increasing phase *11 ^th^ June – 21 ^st^ October*	28,026	28,184	56,210	0.99:1
Wave 2, decreasing phase *22 ^nd^ October–8 ^th^ December*	18,544	20,206	38,750	0.92:1
**Total *11 ^th^ June – 8 ^th^*** ***December***	**46,570**	**48,390**	**94,960**	**0.96:1**

## Discussion/conclusions

The principal finding from this study illustrates that in Ireland the true prevalence of the scale of the COVID-19 epidemic may be twice that which has been recorded through testing. Results for the period from early June 2020 to early December 2020 suggest that the while the prevalence of known cases was approximately 48,000, the asymptomatic prevalence was estimated to be approximately a further 46,000 cases. Furthermore, a detailed analysis of the known number of cases illustrated that as of early December 2020 Ireland has experienced two COVID-19 epidemic waves. The second wave involved almost twice the numbers of cases as the first. Within the first wave most infections occurred among those aged 65 years and older. The age profile of the second wave was significantly different to the first and most cases were observed within those under the age of 25 years.

Results presented must be interpreted in light of their limitations. Reported numbers presented were not adjusted for potential reporting delays. In addition, results of the back-calculation method were computed solely for an incubation period described by a Gamma distribution and other distributions may be equally as applicable. It is also possible that some individuals may have been symptomatic and infectious and did not seek a test and were therefore not recorded in the data. For these reasons are estimate can be considered a minimum estimate.

However, given these limitations the results presented do provide new and additional knowledge on the scale of asymptomatic prevalence within Ireland. Given the estimates of the asymptomatic prevalence during the second wave, and given that known cases are significantly younger than previously, and according to one study directly related to increases in the movement of people ^5^ there is a clear need to focus on transmission between and more importantly from those in younger age groups. The impact of mixing patterns on the spread of disease from one age group to another is well established and it is known that mixing *between* age groups carries far greater risk to the spread of disease than mixing *within* age groups ^19^. It is these mixing patterns which need to be addressed while Ireland awaits vaccine role out and avoids a potential third wave of a COVID-19 epidemic. Further research is needed on asymptomatic prevalence within age groups. Additional research illustrating the role of mixing patterns on spread needs to be disseminated to the wider public to adequately inform them how personal modifications in behaviour can contribute to the control of the epidemic. While universally imposed lockdowns and mitigation measures may be essential, personal behavioural mixing choices are powerful protectors.

## Data availability

Publicly available data was accessed from Our World in Data webpage: https://ourworldindata.org/coronavirus-data and the Health Protection and Surveillance Centre (HPSC) Computerised Infectious Disease Reporting (CIDR) system and available at website https://covid-19.geohive.ie/datasets/d8eb52d56273413b84b0187a4e9117be_0
