# Supplementary material for: The second wave: estimating the hidden asymptomatic prevalence of COVID-19 in Ireland as we plan for imminent immunisation
Source: HRB Open Res. 2022 Feb 25;4:19. Originally published 2021 Feb 18. [Version 2] doi: 10.12688/hrbopenres.13206.2 (PMC8886167; doi:10.12688/hrbopenres.13206.2)
Supplement: Peer review history

# Peer review: Reviewer response for version 2

**DOI:** 10.21956/hrbopenres.14734.r31567

**Published:** 2022-02-28

**Authors:** Harapan Harapan

**Affiliations:** 1 Medical Research Unit, School of Medicine, Universitas Syiah Kuala, Banda Aceh, Indonesia

The authors have responded and addressed my concerns adequately.

Is the work clearly and accurately presented and does it cite the current literature?

Yes

If applicable, is the statistical analysis and its interpretation appropriate?

I cannot comment. A qualified statistician is required.

Are all the source data underlying the results available to ensure full reproducibility?

Yes

Is the study design appropriate and is the work technically sound?

Partly

Are the conclusions drawn adequately supported by the results?

Yes

Are sufficient details of methods and analysis provided to allow replication by others?

Partly

Reviewer Expertise:

Public Health and Virology

I confirm that I have read this submission and believe that I have an appropriate level of expertise to confirm that it is of an acceptable scientific standard.

# Peer review: Reviewer response for version 1

**DOI:** 10.21956/hrbopenres.14356.r30899

**Published:** 2022-01-13

**Authors:** Harapan Harapan

**Affiliations:** 1 Medical Research Unit, School of Medicine, Universitas Syiah Kuala, Banda Aceh, Indonesia

1. It is not clear what does back-calculation method in this study. Should be explained more.
2. What was the justification to divide the wave into two phases?
3. Please also discuss the possibility that some of the individuals might be infected, symptomatic but did not seek healthcare and therefore not reported in the database. 

Is the work clearly and accurately presented and does it cite the current literature?

Yes

If applicable, is the statistical analysis and its interpretation appropriate?

I cannot comment. A qualified statistician is required.

Are all the source data underlying the results available to ensure full reproducibility?

Yes

Is the study design appropriate and is the work technically sound?

Partly

Are the conclusions drawn adequately supported by the results?

Yes

Are sufficient details of methods and analysis provided to allow replication by others?

Partly

Reviewer Expertise:

Public Health and Virology

I confirm that I have read this submission and believe that I have an appropriate level of expertise to confirm that it is of an acceptable scientific standard, however I have significant reservations, as outlined above.

# Response

**Published:** 2022-02-11

**Authors:** Catherine Comiskey

**Affiliations:** Trinity College Dublin, The University of Dublin, Ireland

Thank you for your review and suggestions for improvements.

 In response, I can confirm we have provided a further explanation of the back-calculation method. We have included the text: 

 Working with observed symptomatic cases and the known incubation period, these models predict backwards in time through the incubation period distribution the total numbers of infected and asymptomatic cases these observed cases arose from.

 In response to your comment on the phases within the epidemic waves, we have included the text:

 As two epidemic waves were observed in the data, we study each wave separately. To improve the fit of the models to the data, we fit the models separately to the increasing and decreasing part of these waves.

 In response to your comment on symptomatic cases not being reported in the data we have included within the limitations section the text:

 It is also possible that some individuals may have been symptomatic and infectious and did not seek a test and were therefore not recorded in the data. For these reasons our estimates can be considered a minimum estimate.

 Thank you.

# Peer review: Reviewer response for version 1

**DOI:** 10.21956/hrbopenres.14356.r28936

**Published:** 2021-03-26

**Authors:** Yoon Hong Choi

**Affiliations:** 1 Statistics and Modelling Economics Department, Public Health England, London, UK

Authors estimated the hidden asymptomatic prevalence of COVID-19 in Ireland using the known symptomatic cases and the back-calculation method. Their study is very interesting, important, and timely as the outcomes would provide the policy decision making bodies and public with valuable epidemiological information to implement various control measures to reduce the spread of SARS-CoV-2 in Ireland.

 I have few comments and questions to authors:

1. The definition of the hidden prevalence being estimated is not clear. Authors wrote the following statement:
**Infection we know is spread by both known cases and hidden, undetected asymptomatic cases.**
  It is not clear whether the result of the back-calculation is unknown asymptomatic cases or unknown both symptomatic and asymptomatic cases. Many of symptomatic cases are still not notified to the surveillance due to lack of testing facility availability or self-isolation due to close contacts or only having mild symptoms (no need to use hospital facility).
2. Justification of applying the back calculation method to COVID-19.
  Since the asymptomatic cases do not develop any clinical symptoms, asymptomatic infections would be cleared without developing any symptoms after the incubation period unlike HIV/AIDS. Any comment on this issue would be helpful to justify using this method for the COVID-19 to estimate asymptomatic cases or prevalence.
3. The detection rate on symptomatic cases by different time periods was very dependent on the testing priority and facility availability of SARS-CoV-2 in Ireland.
  This testing facility was poorly available during the first wave and the priority testing was given to only limited people such as who are in long-term care facilities and those with multiple major symptoms in Ireland. This prioritisation could force testing results to detect mainly older adults and very limited number in younger population. Authors need to mention about this time-dependent testing prioritisation and age dependency in different time periods before they suggested that mitigation and prevention measures during the second wave be responsible for the change of the dynamics.
4. Are there any seroprevalence studies to evaluate the estimations in this manuscript?
  Those studies showing antibody levels in the population would be an invaluable measure to evaluate the outcomes of this study as the vaccination was not introduced during the second wave.
5. Authors only present the estimation results during the second wave as the title described. 
  Could you also show the results in the first wave as well? Or, is there any particular reason if not performed?
6. The objective authors mentioned at the end of the introduction is to provide recommendations for future research to enable effective immunisation modelling and planning.
  It would be helpful for readers if authors discuss this matter with their findings.
7. It would be interesting to see authors’ view on the impact of various control measures implemented by the Irish government and human behaviour change in different time periods during this pandemic.
8. It would be interesting to measure hidden symptomatic infections using the fatal cases.
  There is unignorable uncertainty around the “known” symptomatic infections during different periods through COVID-19. As the infection fatality rate became more measurable during the second wave, It might be interesting to estimate the overall number of symptomatic cases using this infection fatality rate and average time to death from infection.

Is the work clearly and accurately presented and does it cite the current literature?

Yes

If applicable, is the statistical analysis and its interpretation appropriate?

Yes

Are all the source data underlying the results available to ensure full reproducibility?

Partly

Is the study design appropriate and is the work technically sound?

Yes

Are the conclusions drawn adequately supported by the results?

Partly

Are sufficient details of methods and analysis provided to allow replication by others?

Yes

Reviewer Expertise:

Mathematical modelling in infectious diseases, epidemiology.

I confirm that I have read this submission and believe that I have an appropriate level of expertise to confirm that it is of an acceptable scientific standard.
